# Protective Effects of Sodium (±)-5-Bromo-2-(α-Hydroxypentyl) Benzoate in a Rodent Model of Global Cerebral Ischemia

**DOI:** 10.3389/fphar.2017.00691

**Published:** 2017-09-27

**Authors:** Yuan Gao, Miao Li, Yan Wang, Zhengqi Li, Chenyu Fan, Zheng Wang, Xinyu Cao, Junbiao Chang, Hailing Qiao

**Affiliations:** ^1^Institute of Clinical Pharmacology, Zhengzhou University, Zhengzhou, China; ^2^College of Chemistry and Molecular Engineering, Zhengzhou University, Zhengzhou, China

**Keywords:** brozopine, global cerebral ischemic rat, neurological function, apoptosis, survival

## Abstract

The aim of the current study was to explore the protective effects of sodium (±)-5-bromo-2-(α-hydroxypentyl) benzoate (brand name: brozopine, BZP) in a rat model of global cerebral ischemia. The rat model was established using a modified Winocur’s method; close postoperative observation was conducted at all times. Neurological function was detected through prehensile traction and beam-walking test. BZP reduced mortality and prolonged the survival time of rats with global cerebral ischemia, within 24 h. There was a decreased survival rate (60%) in the Model group, while the survival rate of the BZP (3 and 12 mg/kg) remarkably increased the survival rate (to 80 and 90%, respectively), in a dose-dependent manner. Compared with the Model group (survival time: 18.50 h), the administration of BZP (0.75, 3, and 12 mg/kg) prolonged the survival time (to 20.38, 21.85, and 23.90 h, respectively), particularly in BZP 12 mg/kg group (*P* < 0.05). Additionally, the BZP (12 mg/kg) group exhibited an improvement in their motor function (*P* < 0.05). The BZP groups (0.75, 3, and 12 mg/kg) displayed significantly reduced necrosis and the percentage of apoptotic cells (*P* < 0.05 and *P* < 0.01, respectively). Compared with Model group, BZP (0.75, 3, and 12 mg/kg) increased the NeuN optical density values (*P* < 0.01). Rats with global ischemia had a high expression of Cyt-c, caspase-3, and the Bax/Bcl-2 ratio compared with sham group (*P* < 0.01). BZP (0.75, 3, and 12 mg/kg), however, reduced the expression of Cyt-c, caspase-3, and the Bax/Bcl-2 ratio, in a dose-dependent manner (*P* < 0.01). There was low expression of p-Akt and PI3K in Model group, compared with the sham group (*P* < 0.01). Meanwhile, BZP (0.75, 3, and 12 mg/kg) increased the expression of p-Akt and PI3K in a dose-dependent manner (*P* < 0.01). We also found the expression of Cyt-c, caspase-3, Bax/Bcl-2 ratio, PI3K, p-Akt, and comprehensive score were directly related. In conclusion, BZP had therapeutic potential and prevented stroke in rat model of global cerebral ischemia. The underlying mechanisms may be related to the inhibition of apoptosis and activation of the survival-signaling-pathway.

## Introduction

Sodium (±)-5-bromo-2-(α-hydroxypentyl) benzoate (BZP), which is derived from 1-3-*n*-butylphthalide (NBP), has a chemical structure that is similar to aspirin. NBP was developed as an anti-cerebral ischemic agent in 2002, in China. Although it was widely used, with good clinic results, increasing reports of adverse reactions such as coagulopathy, gastrointestinal irritation, and liver dysfunction, led to its discontinuation. We designed and synthesized a series of NBP derivatives. The activities of all compounds have been evaluated *in vitro* (Wang et al., 2010). Our previous studies demonstrated that 3-butyl-6-bromo-1(3H)-isobenzofuranone (Br-NBP) had anti-hydrogen peroxide-induced damage in PC12 cells and anti-platelet aggregation effect *in vitro* or *in vivo* on rats (Gao et al., 2010; Ma et al., 2012). Based on our previous findings, BZP played a neuroprotective role against focal cerebral ischemia-reperfusion injury in rats, via anti-apoptosis and anti-inflammation mechanisms, and the promotion of synaptic plasticity (unpublished data). Currently, BZP is used in Phase I clinical trials with encouraging efficacy results. The preventive and therapeutic effects of BZP on a rat model of global ischemia injury have not yet been studied. In this context, we investigated the modulatory effects of BZP on rats following global ischemia injury.

Ischemia injury of brain tissue triggers many cellular complex mechanisms, including excitotoxicity, depolarization, oxidative stress, inflammation, and apoptosis, in addition to many other physiological and pathological processes (Ayuso et al., 2017; de la Tremblaye et al., 2017). Apoptosis is one of the major pathways associated with the activation of a genetic program in which apoptosis effector genes promote cell death, while repressor genes enhance cell survival in cerebral ischemia injury (Yin et al., 2017). Terminal deoxynucleotidyl transferase-mediated dUTP nick end labeling (TUNEL) staining detects DNA fragmentation, which is one of the hallmarks of apoptosis. Two important groups of proteins in the apoptotic cascades are members of the B-cell lymphoma-2 (Bcl-2) family and classes of caspases (Kim et al., 2017). The Bcl-2 family can be classified into the following functionally distinct groups: anti-apoptotic proteins and pro-apoptotic proteins. Bcl-2, an anti-apoptotic protein, regulates apoptotic pathways and protects against cell death. Bcl-2-associated X protein (Bax), a pro-apoptotic protein of the Bcl-2 family that is highly and selectively during apoptosis, promotes cell death (Pena-Blanco and Garcia-Saez, 2017). Caspase-3 is one of the key executors of apoptosis, and the activation of caspase-3 is implicated in apoptotic neuronal cell death in animal models of stroke (Hwang et al., 2013). The rapid translocation of Bax during cerebral ischemia activates the release of cytochrome c (Cyt-c) from the mitochondria, which activates the caspase cascade, leading to apoptosis. The phosphatidylinositol-3 kinase (PI3K)/Akt pathway is a central mediator in signal transduction pathways involved in cell growth, cell survival, and metabolism. It plays a pivotal role in the defense against various neuronal-damaging insults (Wang et al., 2014; Ju et al., 2015; Yang et al., 2015).

## Materials and Methods

### Chemicals

BZP and Br-NBP were synthesized at the Department of Chemistry, Zhengzhou University. The purities of the compounds were 99.4 and 99.8%, respectively. Potassium 2-(1-hydroxypentyl)-benzoate (NBP-K) was purchased from the National Institutes for Food and Drug Control, China. Hematoxylin and eosin (HE) were purchased from Beijing Zhongshan Co., China. Anti-NeuN antibody was purchased from Millipore Co., United States. Mouse monoclonal antibody to Bax, rabbit polyclonal antibody to Bcl-2, rabbit monoclonal antibody to caspase-3, rabbit anti-mouse immunohistochemistry kits to Cyt-c, rabbit polyclonal antibody to PI3K, rabbit polyclonal antibody to p-Akt and TUNEL kits were all purchased from Wuhan Boster Biological Technology Co., China.

### Animals

A total of 70 male Sprague Dawley rats (7 weeks old, weighing 220–300 g) were purchased from the Laboratory Animal Center of Henan province. The animals were housed under controlled environmental conditions (lights on from 6:00 am to 6:00 pm, temperature: 24–26°C, relative humidity: 50–60%) and allowed access to a commercial rat chow and tap water *ad libitum*. The animals were allowed to adapt to the environment for at least 1 week. The rats were fasted 12 h prior to the experiments. All animal experiments were approved by the institutional guidelines of the Experimental Animal Center of the Chinese Academy of Medical Science (certification number: SCXK 2010-0002).

### Global Ischemia Model

The rats were anesthetized using 10% chloral hydrate, fixed on the constant temperature mouse board, and the body temperature was maintained at 36.5∼37.5°C. Modified Winocur’s (Winocur et al., 2013) two vessel blocking method of acute global ischemia model (two-vessel occlusion, 2VO), which were established by permanent ligation of bilateral common, internal, and external carotid arteries (six-vessel occlusion, 6VO) (Li et al., 2015). Sham animals received only separate exposure bilateral common carotid artery, external carotid artery, internal carotid artery, which were not ligated or cut.

### Experimental Setup

**FIGURE 1:**
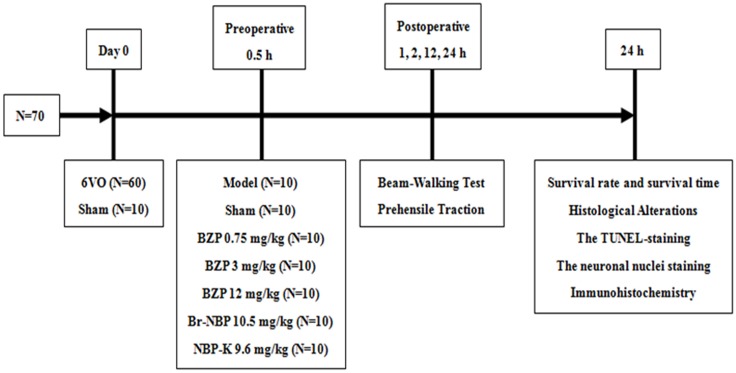
The study design.

Once the ischemic model was established, the rats were divided into the following seven groups: sham group, model group, BZP-treated group (0.75, 3, and 12 mg/kg/day), Br-NBP (10.5 mg/kg/day), and NBP-K group (9.6 mg/kg/day, *n* = 10). The sham group and model group received daily intravenous doses of the same amount of normal saline and other groups were administered the corresponding drug in the same way. Among these groups, BZP 12 mg/kg, Br-NBP 10.5 mg/kg, and NBP-K 9.6 mg/kg were equimolar doses. According to the design of the study, as presented in **Figure 1**.

### Mortality

The postoperative behavioral performances and number of deaths were observed and recorded. Following death, the rat’s brain tissues was analyzed for the relevant indicators so that the ischemic injuries could be described quantitatively.

### Behavioral Assessment

The behavioral assessment was performed under standard laboratory conditions (temperature: 22–24°C and relative humidity: 45–50%) by a research who was blinded to the rats’ experimental groups after 1, 4, 12, and 24 h post-operation.

#### Beam-Walking Test (Urakawa et al., 2007)

Rats were trained to walk on a wooden beam (2.5 cm × 2.5 cm × 80 cm), which was elevated 60 cm above the floor, to reach their home cage. Their performance was evaluated using a modified scale: score 0, the rat traversed the beam with no foot slip; score 1, the rat traversed while grasping the lateral side of the beam; score 2, the rat showed walking disability on the beam but could traverse it; score 3, the rat took a considerable amount of time to traverse the beam due to difficulty walking; score 4, the rat was unable to traverse the beam; score 5, the rat displayed difficulty in moving the body or any limb on the beam; score 6, the rat was unable to stay on the beam for 10 s.

#### Prehensile Traction Test (Hattori et al., 2000)

Rats were trained to grab a wire (with a diameter and the length 0.15 and 100 cm, respectively). The wire was elevated 70 cm above the floor and placed over a 5-cm-thick foam pad in case the rats fell. After placing the front paws on the wire, we recorded the time latency until the rat lost hold of the wire. The performance was evaluated on a four-grade score: score 0, hanging on the wire over 5 s and the hind legs placed on the ropes; score 1, hanging on the wire over 5 s; score 2, hanging on the wire for 3–4 s; score 3, hanging on the wire for 0–2 s.

### Immunohistochemistry

Following the behavioral assessment test, the rats’ brains were removed and immersed in 4% paraformaldehyde with 1 M phosphate buffer, for 48 h. Therefore it was embedded in paraffin, and five groups of 4- to 6-μm-thick sections were prepared for immunostaining and incubated with primary antibodies against Cyt-c, caspase-3, Bax/Bcl-2, p-Akt, or PI3K, followed by incubation with the appropriate secondary antibody.

### Statistical Analysis

Graphs were generated using GraphPad Prism 5 (GraphPad Software, La Jolla, CA, United States). The Student’s unpaired *t*-test, and one-way analysis of variance with Dunnett’s *post hoc* test were performed to statistical analyze of the data. Statistical significance was set at criterion *P* < 0.05. The data are presented as the mean ± standard deviation.

## Results

### BZP Increased the Survival Rate, Prolonged the Survival Time in a Rat of Global Cerebral Ischemia within 24 h

**FIGURE 2:**
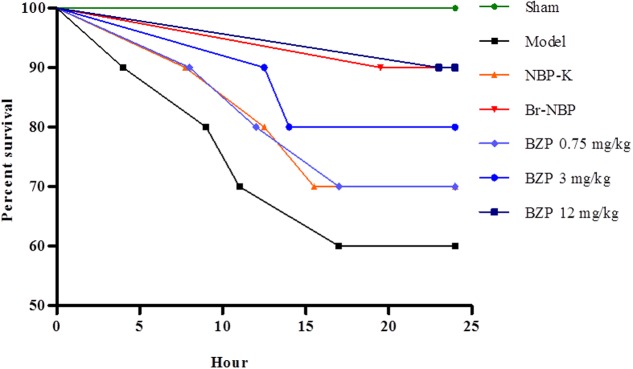
The effect of BZP, within 24 h, on survival rate and survival time in a rat model of global cerebral ischemia. The *x*-axis represents the hour of administration.

The global cerebral ischemia model was established using the modified Winocur’s method on rats. Close postoperative observation was maintained at all times. As shown in **Figure 2**, 24 h later, there was a decreased survival rate (60%) in the Model group, while the survival rate of the BZP (3 and 12 mg/kg) remarkably increased the survival rate (to 80 and 90%, respectively), in a dose-dependent manner. Compared with the model group (survival time: 18.50 h), the administration of BZP (0.75, 3, and 12 mg/kg) prolonged the survival time (to 20.38, 21.85, and 23.90 h, respectively), particularly in BZP 12 mg/kg group (*P* < 0.05). These results demonstrated that BZP increased the survival rate and prolonged the survival time in rats with global ischemia.

### Behavioral Testing

**FIGURE 3:**
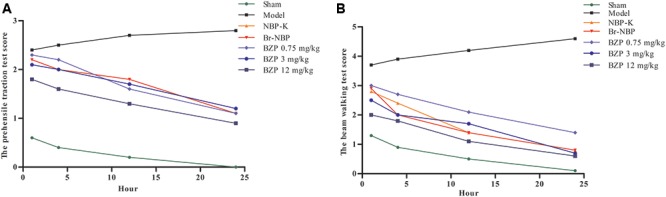
The neurological deficits score on pre-ischemic treatment with BZP in rats with global ischemia. **(A)** Prehensile traction test. **(B)** Beam-walking test. The *x*-axis represents the hour of administration, while the *y*-axis represent the prehensile traction score and beam-walking score. N^∗^ is the number of survival animals after 24 h.

Following BZP administration, the rat’s prehensile traction and beam-walking ability were assessed to directly investigate the effect of BZP on functional recovery. Compared with the sham group, which exhibited an intact performance, the cerebral lesions in the rats with global cerebral ischemia caused behavioral deficits, as evidenced by a reduced time on the beam in the beam-walk test. In contrast, rats in the BZP groups remained on the beam for longer, compared with the Model group. Moreover, in the prehensile traction test, compared with the Model group, rats that were treated with various doses of BZP spent significantly more time on the rope an increase was observed in rats treated with different doses of BZP. There were significant differences in the time spent on the rope (*P* < 0.05). Additionally, the BZP (12 mg/kg) group exhibited an improvement in their motor function, which was more prevalent than in the NBP-K (9.6 mg/kg) and Br-NBP (10.5 mg/kg) groups. A significant group effect in the grip strength was observed at hours 4, 12, and 24, while rats treated with BZP (0.75, 3, and 12 mg/kg) achieved significantly lower scores than the other experimental groups (**Figure 3**).

### Histological Alterations

**FIGURE 4:**
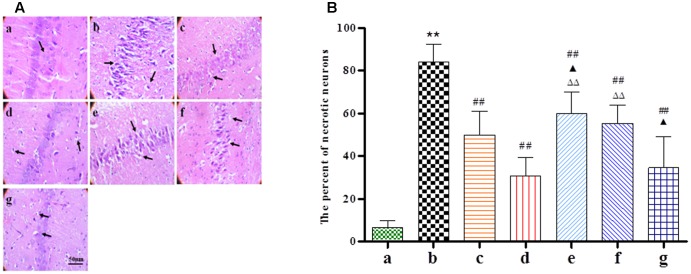
The effect of BZP on morphologic changes in the CA1 region of the hippocampus in rats with global ischemia. The total number of necrosis cells was counted in five distinct areas of the hippocampus under the microscopic field (magnification ×200) in each group. **(A,B)** The different groups are as follows: (a) Sham, (b) Model group, (c) NBP-K (9.6 mg/kg), (d) Br-NBP (10.5 mg/kg), and (e–g) BZP (0.75, 3, and 12 mg/kg, respectively). Data were presented as mean ± standard deviation. A one-way analysis of variance was used to determine if the differences between the groups were statistically significant. ^∗∗^*P* < 0.01 *vs* Sham group; ^#^*P* < 0.05, ^##^*P* < 0.01 *vs* Model group; ^
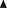
^*P* < 0.05, ^
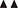
^*P* < 0.01 *vs* NBP-K group; ^
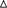
^*P* < 0.05, ^
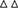
^*P* < 0.01 *vs* Br-NBP group. Arrows show that nerve cells sparse, irregular, even disappeared, nucleus shrinkage with neuronal necrosis.

HE staining was performed at 24 h after BZP administration to observe its effect on the morphology of hippocampal neurons. As shown in **Figure 4**, typical neuropathological changes, including neuronal loss and nucleus shrinkage or disappearance were observed in the CA1 of the hippocampus in Model group. Further, there was a reduction in the density of healthy neuron cells in the CA1 region in the Model group compared with the sham group (*P* < 0.01). The BZP groups (0.75, 3, and 12 mg/kg) displayed significantly reduced necrosis (*P* < 0.05 and *P* < 0.01, respectively).

### BZP Inhibited Neuronal Apoptosis in a Rat Model of Global Ischemia

**FIGURE 5:**
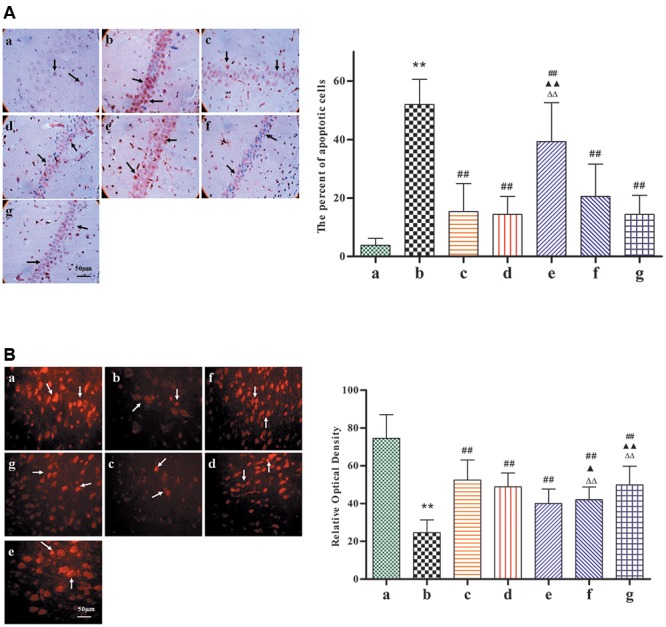
Images of the expression of neuronal nuclei and apoptosis, following pretreatment with BZP, in ischemia-induced rats. Total number of apoptotic cells and neuronal nuclei was counted in five distinct areas of the ischemic penumbra under a microscopic field (magnification × 200), in each group. **(A)** TUNEL-staining. **(B)** Neuronal nuclei staining. The different groups are as follows: (a) Sham, (b) Model group, (c) NBP-K (9.6 mg/kg), (d) Br-NBP (10.5 mg/kg), and (e–g) BZP (0.75, 3, and 12 mg/kg, respectively). Data were presented as mean ± standard deviation. A one-way analysis of variance was used to determine if the differences were statistically significant. ^∗∗^*P* < 0.01 *vs* Sham group; ^#^*P* < 0.05, ^##^*P* < 0.01 *vs* Model group; ^
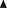
^*P* < 0.05, ^
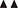
^*P* < 0.01 *vs* NBP-K group; ^
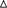
^*P* < 0.05, ^
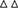
^*P* < 0.01 *vs* Br-NBP group. Arrows in **(A)** show that cell body shrinkage, nuclear pyknosis, nuclear fragmentation, and the formation apoptotic bodies. Arrows in **(B)** show that mature neurons loss.

TUNEL staining reveals apoptosis-positive cells, which have the following features: cell body shrinkage, nuclear pyknosis, nuclear fragmentation, and the formation apoptotic bodies. In comparison with Model group, as shown in **Figure 5A**, the percentage of apoptotic cells had significantly decreased with BZP (0.75, 3, and 12 mg/kg; *P* < 0.01). In addition, NeuN-expressing mature neurons were absent in the brain tissue after the induction of global ischemia. Compared with model group, as shown in **Figure 5B**, BZP (0.75, 3, and 12 mg/kg) increased the NeuN optical density values (*P* < 0.01). BZP (12 mg/kg) was better than NBP-K group (*P* < 0.01) and Br-NBP group (*P* < 0.01).

### BZP Decreased the Expression of Cyt-c, Caspase-3, the Ratio of Bax/Bcl-2, and Elevated the Level of p-Akt and PI3K in the CA1 Hippocampal Region in Rats with Global Ischemia Injury

**FIGURE 6:**
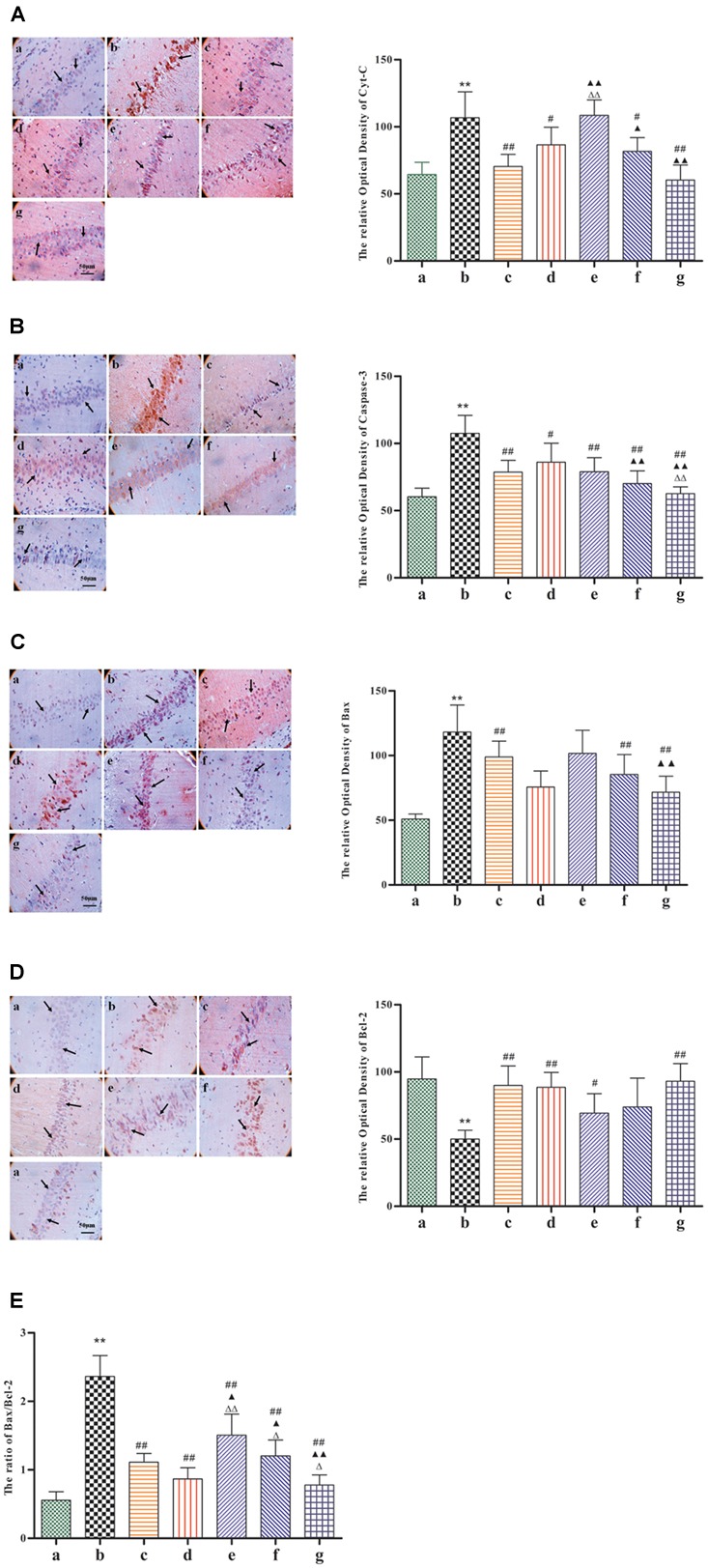
Immunohistochemical staining analysis of Cyt-c, caspase-3, the Bax, and Bcl-2 ratio in the CA1 hippocampal region in rats with global cerebral ischemia, after pretreatment with BZP, NBP-k, or Br-NBP. The total number of positive cells was counted in five distinct areas of the ischemic penumbra under the microscopic field (magnification ×200) in each group. **(A)** Cyt-c. **(B)** Caspase-3. **(C)** Bax. **(D)** Bcl-2. **(E)** The Bax/Bcl-2 ratio. The different groups are as follows: (a) Sham, (b) Model group, (c) NBP-K (9.6 mg/kg), (d) Br-NBP (10.5 mg/kg), and (e–g) BZP (0.75, 3, and 12 mg/kg, respectively). Data were presented as mean ± standard deviation. A one-way analysis of variance was used to determine if the differences between groups were statistically significant. ^∗∗^*P* < 0.01 *vs* Sham group; ^#^*P* < 0.05, ^##^*P* < 0.01 *vs* Model group; ^
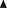
^*P* < 0.05, ^
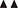
^*P* < 0.01 *vs* NBP-K group; ^
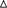
^*P* < 0.05, ^
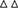
^*P* < 0.01 *vs* Br-NBP group. Arrows show that the expression of Cyt-c, caspase-3, Bax, Bcl-2, p-Akt and PI3K positive cell.

**FIGURE 7:**
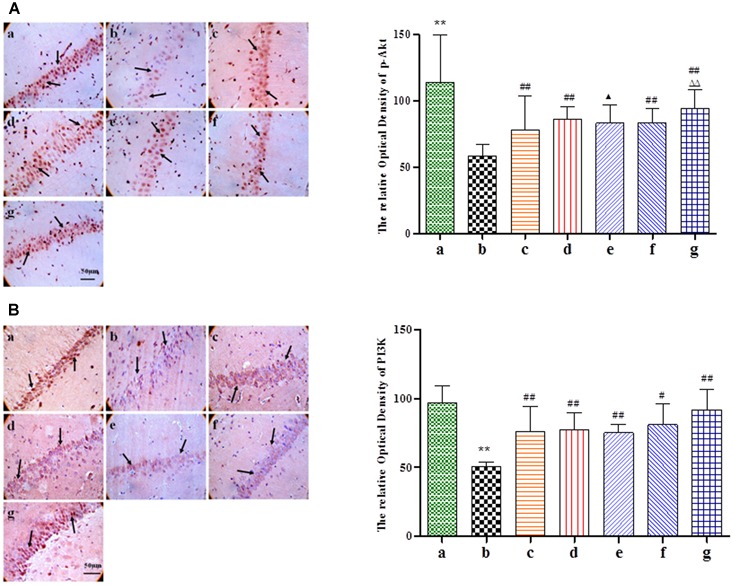
Immunohistochemical staining analysis of p-Akt and PI3K in the CA1 hippocampus region of rats with global cerebral ischemia, after pretreatment with BZP, NBP-K, or Br-NBP. The total number of positive cells was counted in five distinct areas of ischemic penumbra under the microscopic field (magnification ×200) in each group. **(A)** p-Akt. **(B)** PI3K. The groups were as follows: (a) Sham, (b) Model group, (c) NBP-K (9.6 mg/kg), (d) Br-NBP (10.5 mg/kg), and (e–g) BZP (0.75, 3, and 12 mg/kg, respectively). Data were presented as mean ± standard deviation. A one-way analysis of variance was used to determine if the differences between the groups were statistically significant. ^∗∗^*P* < 0.01 *vs* Sham group; ^#^*P* < 0.05, ^##^*P* < 0.01 *vs* Model group; ^
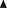
^*P* < 0.05, ^
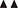
^*P* < 0.01 *vs* NBP-K group; ^
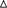
^*P* < 0.05, ^
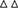
^*P* < 0.01 *vs* Br-NBP group. Arrows show that the expression of Cyt-c, caspase-3, Bax, Bcl-2, p-Akt and PI3K positive cell.

Given its neuroprotective effects, we further investigated the effect of BZP on the mitochondrial signaling pathway following global cerebral ischemia. The activation of genes for apoptosis factors promotes cell death, while repressor genes enhance cell survival. As shown in **Figure 6**, rats with global ischemia had a high expression of Cyt-c, caspase-3, and the Bax/Bcl-2 ratio compared with sham group (*P* < 0.01). BZP (3, 0.75, and 12 mg/kg), however, reduced the expression of Cyt-c, caspase-3, and the Bax/Bcl-2 ratio, in a dose-dependent manner (*P* < 0.01). In addition, BZP 12 mg/kg had a better effect on the expression of Cyt-c, caspase-3, and the Bax/Bcl-2 ratio than NBP-K 9.6 mg/kg (*P* < 0.05, *P* < 0.01) and Br-NBP 10.5 mg/kg (*P* < 0.05, *P* < 0.01). As shown in **Figure 7**, there was low expression of p-Akt and PI3K in Model group, compared with the sham group (*P* < 0.01). Meanwhile, BZP (3, 0.75, and 12 mg/kg) increased the expression of p-Akt and PI3K in a dose-dependent manner (*P* < 0.01).

### Significant Correlation between the Expression of Apoptosis-Related Factors and Comprehensive Score in Rats with Global Ischemia

**FIGURE 8:**
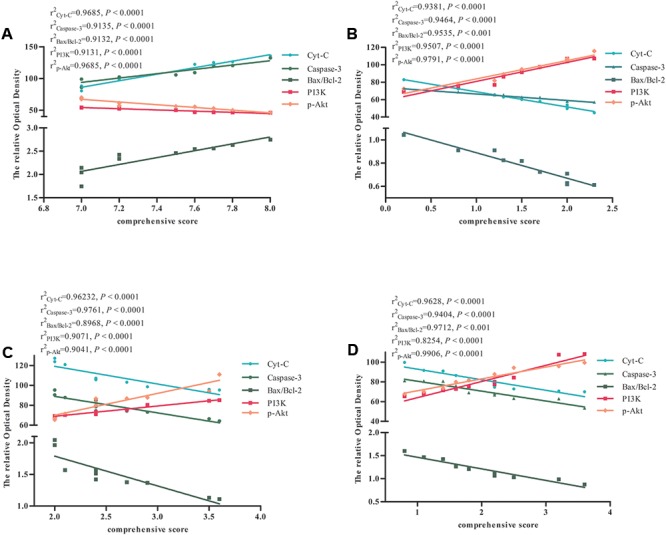
The correlation between the expression of apoptosis-related factors and comprehensive score in Model and BZP (0.75, 3, and 12 mg/kg) groups in rats with global ischemia. Correlation between the expression of apoptosis-related factors and comprehensive score in **(A)** Model group, **(B)** BZP 12 mg/kg group, **(C)** BZP 3 mg/kg group, and **(D)** BZP 0.75 mg/kg group. *r*^2^, correlation coefficient of the expression of apoptosis-related factors and comprehensive score. Statistical significant denoted by *P* < 0.001.

We performed a correlation analysis between the expression of Cyt-c, caspase-3, Bax/Bcl-2 ratio, PI3K, p-Akt, and comprehensive score in the Model and BZP (0.75, 3, and 12 mg/kg) groups. The comprehensive score was calculated by adding the prehensile-traction score to the beam-walking score. As shown in **Figure 8**, the expression of Cyt-c, caspase-3, Bax/Bcl-2 ratio, PI3K, p-Akt, and comprehensive score were directly related. Thus, a higher expression of Cyt-c, caspase-3, and Bax/Bcl-2, was associated with a lower the expression of PI3K and p-Akt and the more severe neurological deficit.

## Discussion

Although BZP was distributed in tissue from both the normal and global cerebral ischemia brains, it was particularly highly concentrated in the latter, where it was mostly metabolized into Br-NBP (data not shown). Interestingly, Br-NBP was an active metabolite, with anti-oxidant stress and anti-platelet aggregation effects. Therefore, Br-NBP was an active metabolic compound that was closely observed. NBP-K was regarded as the pro-drug of NBP and NBP-K was used as a positive control drug in the current study. The anti-ischemic action of BZP was 4–16 times that of Br-NBP or NBP-K, at the equivalent molar dose. Thus, we speculated that the neuroprotective effect of BZP depended on itself and Br-NBP. The potency of BZP was greater than Br-NBP, implying that it had a new chemical structure and was different from NBP-K and Br-NBP. The present study demonstrated the neuroprotective effect of BZP against global cerebral ischemia injury.

A model of global ischemia should closely mimic the main clinical symptoms of the disease. Compared with the traditional 2VO model, the neurological deficit, motor coordination, and pathological damage were more serious in the 6VO model. Therefore, the 6VO model was more suitable as a rat model of global cerebral ischemia, for the current study, in comparison to the 2VO method. The results also highlighted that the administration of BZP (0.75, 3, and 12 mg/kg) and Br-NBP decreased the mortality and prolonged the survival time, particularly at higher doses.

Since global ischemic injury had destroyed the sensory and motor centers of the rat model of the disease, assessing behavioral function recovery allowed the effect of the BZP treatment to be evaluated. The beam-walking test is a common method used to gauge the recovery of sensory and motor function. The prehensile traction test is used to measure the ability of the forepaw and forelimb in rats. In our data, at 24 h, the rats could walk properly in the sham group. Compared with the Model group, the scores of rats in the treatment groups were lower and gradually, decreased over time. This suggested that the motor function was gradually restored. The active function of the rat upper limbs and balance were improved by a high dose of BZP was superior to NBP-K or Br-NBP.

Treatment with BZP also inhibited the mitochondrial apoptotic pathway, and markedly reduced ischemia-induced neuronal apoptotic cells within penumbra, 24 h after global cerebral ischemic injury. Experimental and clinical evidence has demonstrated that cerebral ischemia disrupts the blood–brain barrier permeability (Beker et al., 2015; Hong et al., 2015). The present study provided evidence to confirm that BZP exerted its neuroprotective effects through the inhibition of the mitochondrial apoptotic pathway after transient global cerebral ischemia. Additionally, BZP reduced the number of TUNEL-positive cells and apoptotic bodies, and even enhanced the level of anti-apoptotic protein. Apoptosis is considered the prominent form of neuronal death in the penumbra in stroke (Cheng et al., 2015). The penumbra, which is an area of partially preserved energy metabolism, represents ischemic brain tissue that is functionally impaired, but where the injury is potentially reversible. When considering therapeutic strategies for ischemic stroke, the rescue of the penumbra is a crucial consideration (Chelluboina et al., 2015). There is increasing evidence that the penumbra area of nuclear factor kappa B (NF-κB) activity significantly overlaps with tumor necrosis factor alpha (TNF-α) overproduction and apoptosis. The NF-κB activity and enhanced TNF-α expression could be attributed to an apoptotic pathway following global cerebral ischemia. Apoptosis, which is mediated by mitochondrial disturbances, is a major cause of cellular damage in the ischemic penumbra. The mitochondrial release of apoptogenic factors drives neuronal cell death during cerebral ischemia. Proteins in the proapoptotic Bcl-2 family, such as Bax and BAK, reside in the mitochondrial outer membrane and constituted a gateway to the apoptotic process. Bax and BAK are also located at the endoplasmic reticulum, where they regulated calcium fluxes. Bax, which is homologous to Bcl-2, has antagonistic actions to the latter. Thus, the ratio of these proteins is an index to evaluate the level of cell apoptosis. High levels of calcium in the mitochondria disrupts the mitochondrial outer membrane by increasing permeability transition and promoting Cyt-c release, which acts as a trigger for neuronal apoptosis. Caspases become subsequently activated, resulting in apoptosis (Lazarovici et al., 2012; Li et al., 2012; Jayakumar et al., 2013; Bradley et al., 2014). Caspase-3, in particular, causes degradation of the cytoskeleton, DNA fragmentation, and eventually, cell death. In contrast, anti-apoptotic mediators such as Bcl-2 and Bcl-xL increase cell survival after ischemia by preserving mitochondrial membrane integrity (Chen et al., 2015). The results showed that BZP significantly reduced the expression of Cyt-c and caspase-3 in the ischemic penumbra, while decreasing the ratio of Bax/Bcl-2 in rats with global cerebral ischemia. The strong positive correlation between the changes of NF-κB and other inflammatory proteins corroborated with and validated previous findings. However, we currently do not know why the extent of the correlation between BZP dosages and various inflammatory proteins differ. Further research on detailed anti-inflammatory effects of BZP is currently in progress. Apoptosis is associated with the activation of a genetic program in which apoptosis effector genes promote cell death, while repressor genes enhance cell survival. It has been proven that, in a variety of organisms, the inhibition of the PI3K/p-Akt signaling pathway decreases the rate of cell metabolism, which delays cell senescence (Abdel-Aleem et al., 2016). The inhibition of cell apoptosis is initiated by the activation of PI3K, which activates the upstream kinase, Akt is necessary for the survival of nerve growth factor-dependent sympathetic neurons, which also maintains or enhances the survival of neurons (Crowder and Freeman, 1998). p-Akt can also inhibit the activation of caspase-9. In addition, p-Akt can enter the nucleus and inhibit the phosphorylation of the Forkhead family, thereby blocking the Fas apoptosis pathway (Murphy, 2004; Song et al., 2005). In the current study, BZP increased the expression of PI3K and p-Akt, suggesting that it inhibited apoptosis in rats with global ischemia injury by activating PI3K/p-Akt signaling pathway.

## Conclusion

BZP could be an effective the treatment and prevention of global ischemic stroke. The results of the current study suggest that BZP elicits neuroprotective effects by inhibiting apoptosis and activating neuronal survival. However, further research on a unifying hypothesis is warranted to validate our conclusions.

## Author Contributions

YG, ML, YW, ZL, CF, ZW, XC, JC, and HQ meet the essential authorship criteria required by the journal to be observed including (a) substantial contributions to the conception or design of the work; or the acquisition, analysis, or interpretation of data for the work; and (b) drafting the work or revising it critically for important intellectual content; and (c) final approval of the version to be published; and (d) agreement to be accountable for all aspects of the work in ensuring that questions related to the accuracy or integrity of any part of the work are appropriately investigated and resolved.

## Conflict of Interest Statement

The authors declare that the research was conducted in the absence of any commercial or financial relationships that could be construed as a potential conflict of interest.

## Abbreviations

ASA: aspirin
Bax: Bcl-2-associated X protein
Bcl-2: B-cell lymphoma-2
Br-NBP: 3-butyl-6-bromo-1(3H)-isobenzofuranone
BZP: sodium (±)-5-bromo-2-(α-hydroxypentyl) benzoate, brozopine
Cyt-c: cytochrome c
DNA: deoxyribonucleic acid
HE: hematoxylin and eosin
NBP: 1-3-*n*-butylphthalide
NBP-K: potassium 2-(1-hydroxypentyl)-benzoate
NeuN: neuronal nuclei
NF-κB: nuclear factor kappa B
PI3K: phosphatidylinositol-3 kinase
SD: Sprague Dawley
TNF-α: tumor necrosis factor alpha
TUNEL: terminal deoxynucleotidyl transferase-mediated dUTP nick end labeling
2VO: two-vessel occlusion
6VO: six-vessel occlusion.
